# Supplementary material for: The causal relationship between the human gut microbiota and pyogenic arthritis: a Mendelian randomization study
Source: Front Cell Infect Microbiol. 2024 Nov 26;14:1452480. doi: 10.3389/fcimb.2024.1452480 (PMC11629706; doi:10.3389/fcimb.2024.1452480)
Supplement: Supplementary file 1 [file DataSheet1.zip › mendelian test/ebi-a-GCST90027674.csv_forest.pdf]

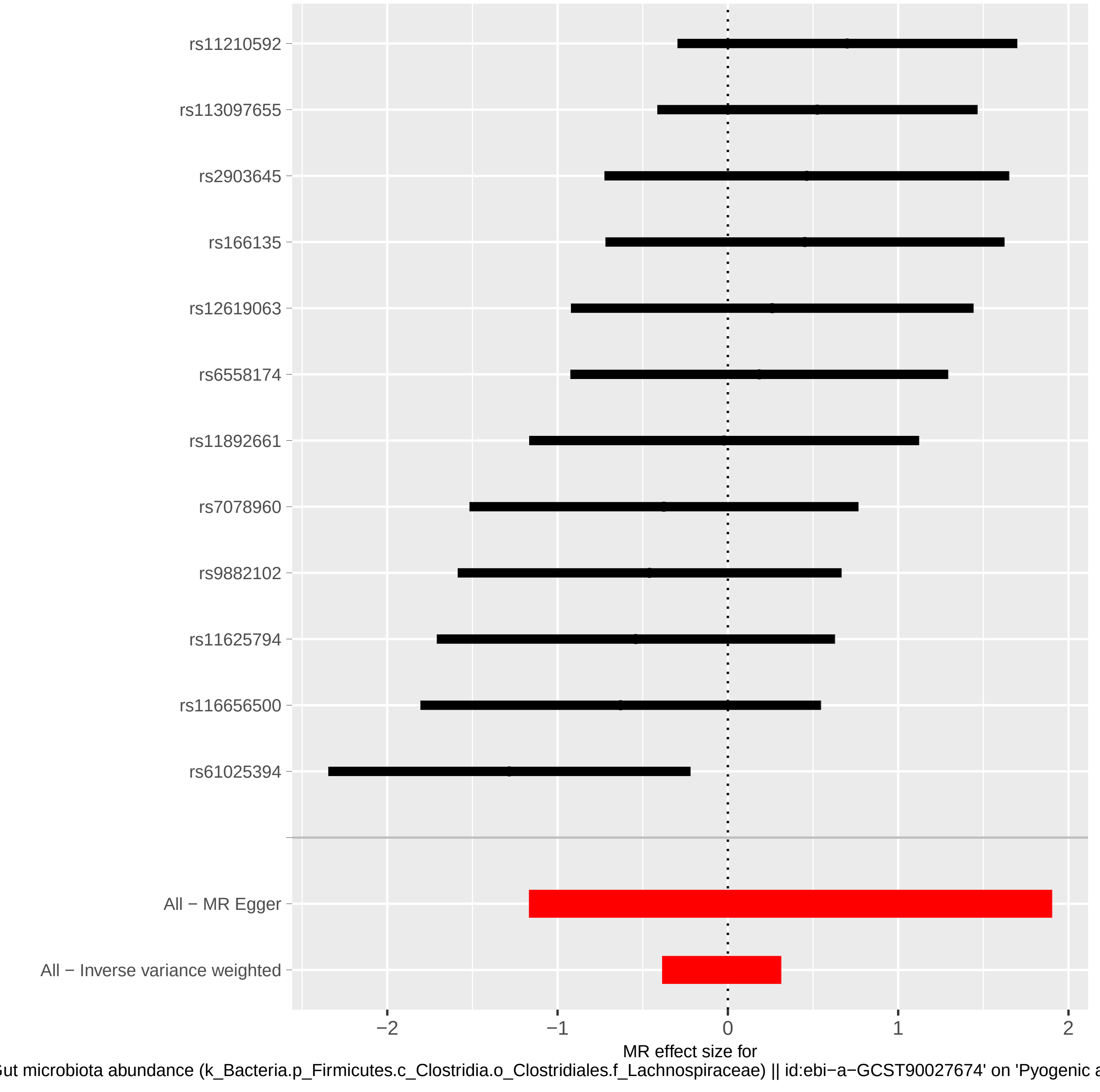
rs11210592
rs113097655
rs2903645
rs166135
rs12619063
rs6558174
rs11892661
rs7078960
rs9882102
rs11625794
rs116656500
rs61025394
All − MR Egger
All − Inverse variance weighted
−2
−1
0
1
2
MR effect size for
Gut microbiota abundance (k_Bacteria.p_Firmicutes.c_Clostridia.o_Clostridiales.f_Lachnospiraceae) || id:ebi−a−GCST90027674' on 'Pyogenic a
